# Supplementary material for: Detecting sample swaps in diverse NGS data types using linkage disequilibrium
Source: Nat Commun. 2020 Jul 29;11:3697. doi: 10.1038/s41467-020-17453-5 (PMC7391710; doi:10.1038/s41467-020-17453-5)
Supplement: Supplementary file 1 — Supplementary Information [file 41467_2020_17453_MOESM1_ESM.pdf]

Supplementary Material

Detecting sample swaps in diverse NGS datatypes using linkage disequilibrium

Nauman Javed[1], Yossi Farjoun[2], Tim J. Fennell[2], Charles B. Epstein[2], Bradley E. Bernstein[1,2], Noam Shoresh[1,2,†]

[1]Department of Pathology and Center for Cancer Research, Massachusetts General Hospital and Harvard Medical School, Boston, Massachusetts 02114, USA.

[2]Broad Institute of MIT and Harvard, Cambridge, Massachusetts 02142, USA.

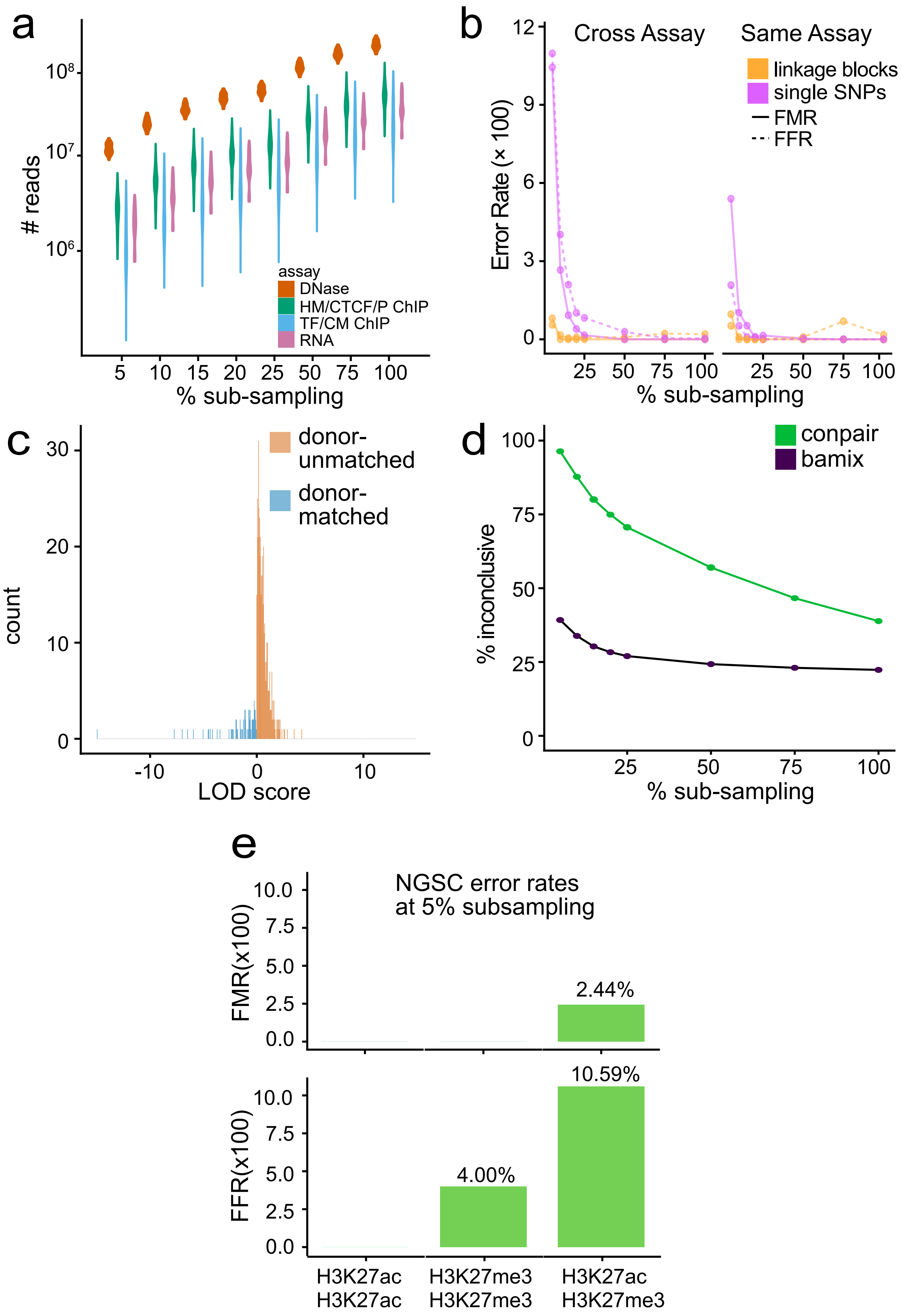

a
reads
10^8
10^7
10^6
5 10 15 20 25 50 75 100
% sub-sampling
assay
DNase
HM/CTCF/P ChIP
TF/CM ChIP
RNA
b
Cross Assay
Same Assay
Error Rate (× 100)
12 9 6 3 0
linkage blocks
single SNPs
FMR
FFR
25 50 75 100
% sub-sampling
c
donor-unmatched
donor-matched
count
30 20 10 0
-10 0 10
LOD score
d
conpair
bamix
% inconclusive
100 75 50 25 0
25 50 75 100
% sub-sampling
e
NGSC error rates
at 5% subsampling
FMR(x100)
10.0 7.5 5.0 2.5 0.0
2.44%
FFR(x100)
10.59%
4.00%
H3K27ac H3K27ac
H3K27me3 H3K27me3
H3K27ac H3K27me3

Supplementary Figure 1. (a) Distribution of number of reads in sub-sampled datasets used for benchmarking, broken down by assay type. ChIP datasets were divided into two classes – those which targeted transcription factor (TF) and chromatin modifier (CM), and those which targeted broad histone modifications (HM), POL2/POL2RA (P), or CTCF. **(b)** Comparison of percentage false match (FM) and false flag (FF) rates for 9767 same-donor and 34336 different donor pairwise comparisons using Crosscheck with either linkage blocks, or single SNPs only. Across different (left) and same (right) assay comparisons, incorporation of linkage information (orange line) decreases the FF and FM percentage, particularly at sub-sampling percentages. Comparisons are classified as *same-assay* if the two datasets are from the same assay type, and have the same target epitope in the case of ChIP-seq datasets. All other comparisons are classified as *cross-assay.* **(c)** Distribution of LOD scores from false flags and false matches from benchmarking experiments. The distribution of the majority (99%) of LOD scores from these misclassifications is used to create an “inconclusive” range of LOD scores, in which donor-match or mismatch cannot be confidently called. **(d)** Percent inconclusive genotype concordance calls for 9767 same-donor and 29573 different donor pairwise comparisons using Conpair and BAMixChecker. “Inconclusive” is defined as pairwise comparisons resulting in genotype concordances between 50 and 80% for Conpair, and a score of 0 for BAMixChecker. **(e)** FMR and FFR for NGSC at 5% subsampling for pairwise comparisons between ChIP-seq datasets targeting the non-overlapping histone modifications H3K27ac and H3K27me3. NGSC performs worse for comparisons between H3K27ac and H3K27me3 datasets (n=41 donor-matched, n=85 donor-mismatched) than for comparisons between two H3K27ac (n=24 donor-matched, n=67) or two H3K27me3 datasets (n=11 donor-matched, n=25 donor-mismatched). In contrast, Crosscheck classifies all pairs correctly.

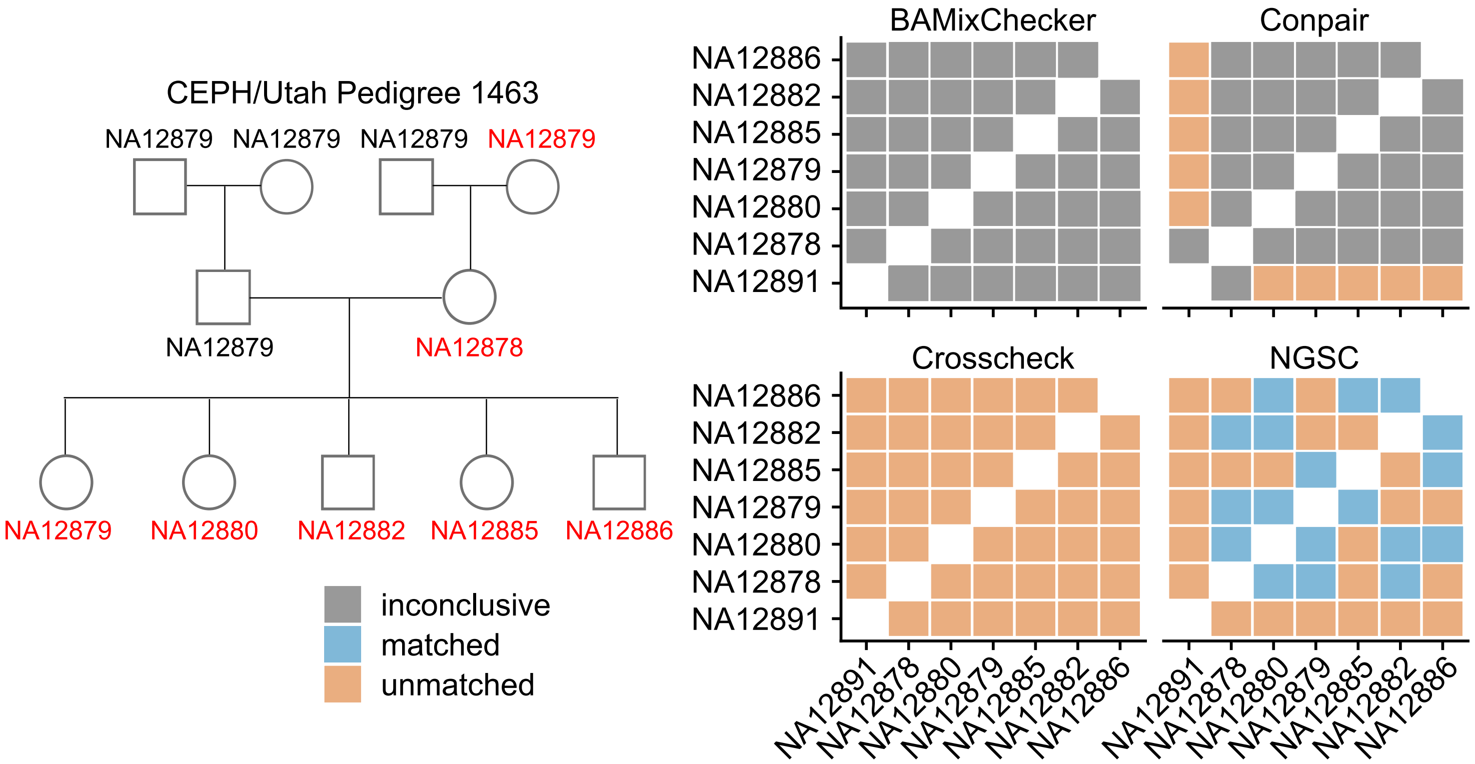

Supplementary Figure 2: Performance of NGSC, Crosscheck, BAMixChecker, and Conpair on familial datasets. Each method was used to classify 21 pairwise comparisons between RNA-seq datasets from 7 related individuals (indicated in red) from CEPH/Utah pedigree 1463. "Inconclusive" is defined as pairwise comparisons resulting in genotype concordance between 50 and 80% for Conpair, a score of 0 for BAMixChecker, and an LOD score between -5 and 5 for Crosscheck. NGSC incorrectly classifies 43% of pairs, while Conpair and BAMixChecker are inconclusive for 76 and 100% of pairs respectively.
In contrast, Crosscheck correctly classifies all dataset pairs as mismatches.

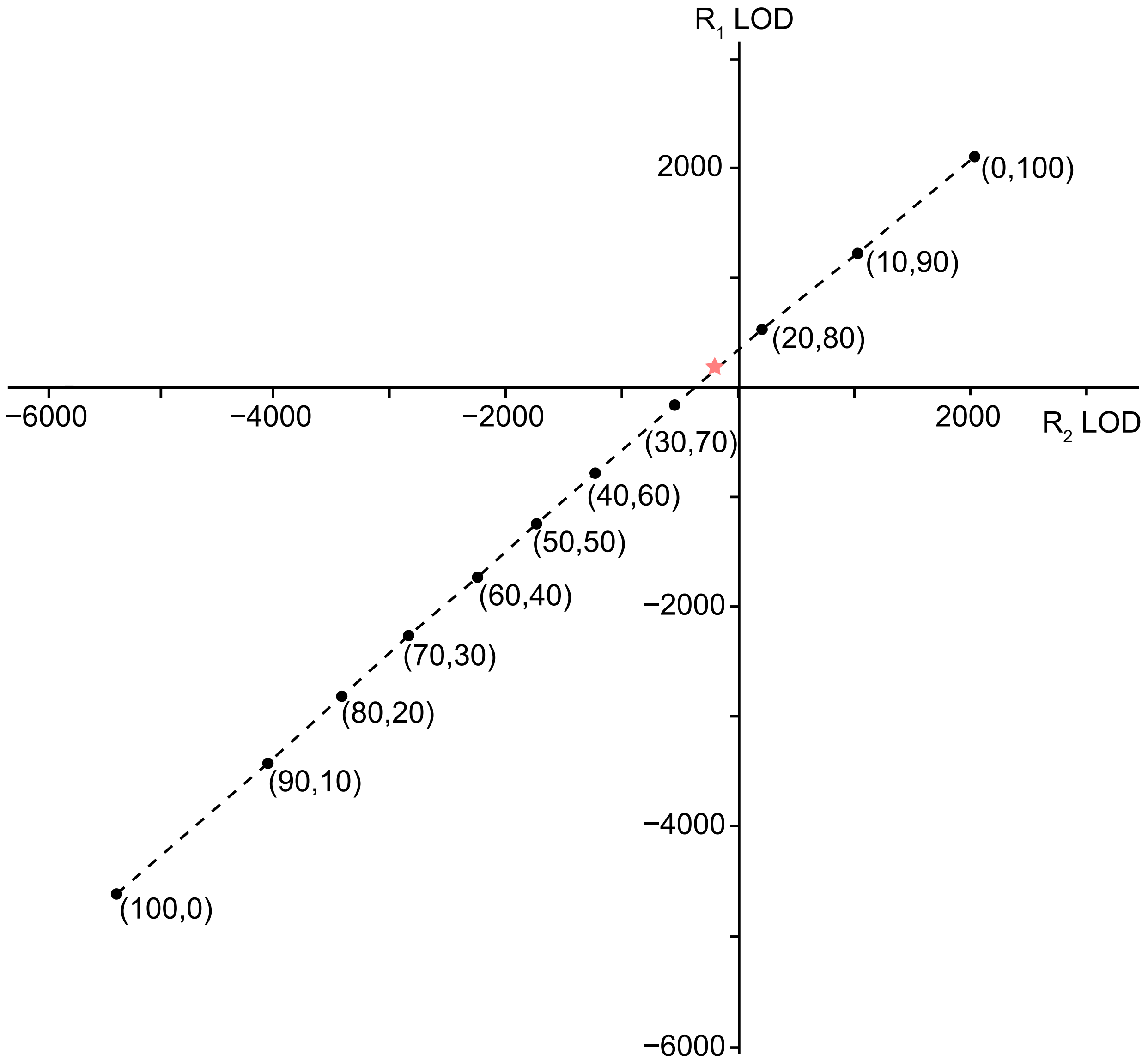

Supplementary Figure 3: Demonstration of Crosscheck's performance for contaminated datasets. Simulated contaminated datasets were created by combining various proportions of two ENCODE ChIP-seq datasets derived from two different donors: ENCFF005HON and ENCFF007DFB. Proportions of reads deriving from ENCFF005HON and ENCFF007DFB respectively are indicated in parentheses for each mixture. Each mixture was compared to two datasets derived from the same donor as ENCFF005HON, ENCFF007NTA (R_1) and ENCFF029GAR (R_2). The star indicates a region where a contaminated sample can score as a donor match against one dataset (R_1), but score as a donor mismatch against a different dataset from the same donor (R_2).
