# Supplementary material for: Detecting sample swaps in diverse NGS data types using linkage disequilibrium
Source: Nat Commun. 2020 Jul 29;11:3697. doi: 10.1038/s41467-020-17453-5 (PMC7391710; doi:10.1038/s41467-020-17453-5)
Supplement: Supplementary file 3 — Descriptions of Additional Supplementary Files [file 41467_2020_17453_MOESM3_ESM.pdf]

Descriptions of Additional Supplementary Files

Supplementary Data 1

Description: Metadata for all files used for benchmarking with verified donor annotations.

Supplementary Data 2

Description: Metadata for all files used for benchmarking without verified donor annotations.

Supplementary Data 3

Description: Metadata for all ENCODE files analysed.
