# Supplementary material for: Detecting sample swaps in diverse NGS data types using linkage disequilibrium
Source: Nat Commun. 2020 Jul 29;11:3697. doi: 10.1038/s41467-020-17453-5 (PMC7391710; doi:10.1038/s41467-020-17453-5)
Supplement: Supplementary file 7 — Reporting Summary [file 41467_2020_17453_MOESM7_ESM.pdf]

nature research

Corresponding author(s): Noam Shoresh

Last updated by author(s): Jun 19, 2020

Reporting Summary

Nature Research wishes to improve the reproducibility of the work that we publish. This form provides structure for consistency and transparency in reporting. For further information on Nature Research policies, see Authors & Referees and the Editorial Policy Checklist.

Statistics

For all statistical analyses, confirm that the following items are present in the figure legend, table legend, main text, or Methods section.

n/a	Confirmed	
☐	☒	The exact sample size (*n*) for each experimental group/condition, given as a discrete number and unit of measurement
☒	☐	A statement on whether measurements were taken from distinct samples or whether the same sample was measured repeatedly
☒	☐	The statistical test(s) used AND whether they are one- or two-sided<br>*Only common tests should be described solely by name; describe more complex techniques in the Methods section.*
☒	☐	A description of all covariates tested
☒	☐	A description of any assumptions or corrections, such as tests of normality and adjustment for multiple comparisons
☒	☐	A full description of the statistical parameters including central tendency (e.g. means) or other basic estimates (e.g. regression coefficient) AND variation (e.g. standard deviation) or associated estimates of uncertainty (e.g. confidence intervals)
☒	☐	For null hypothesis testing, the test statistic (e.g. *F*, *t*, *r*) with confidence intervals, effect sizes, degrees of freedom and *P* value noted<br>*Give P values as exact values whenever suitable.*
☒	☐	For Bayesian analysis, information on the choice of priors and Markov chain Monte Carlo settings
☒	☐	For hierarchical and complex designs, identification of the appropriate level for tests and full reporting of outcomes
☒	☐	Estimates of effect sizes (e.g. Cohen's *d*, Pearson's *r*), indicating how they were calculated

Our web collection on statistics for biologists contains articles on many of the points above.

Software and code

Policy information about availability of computer code

Data collection	No software was used
Data analysis	samtools v1.8, BEDTools v2.26.0, R 3.6, STAR 2.6.0c, GATK 4, Picard-Tools v2(https://github.com/broadinstitute/picard), BAMixChecker, NGSCheckmate, Conpair v0.2, WDL v1.0(Workflow Description Language), PLINK2, fingerprint_maps(https://github.com/naumanjaved/fingerprint_maps), Anaconda v3.8.3, LD SCore v1.0.1

For manuscripts utilizing custom algorithms or software that are central to the research but not yet described in published literature, software must be made available to editors/reviewers. We strongly encourage code deposition in a community repository (e.g. GitHub). See the Nature Research guidelines for submitting code & software for further information.

Data

Policy information about availability of data

All manuscripts must include a data availability statement. This statement should provide the following information, where applicable:

- Accession codes, unique identifiers, or web links for publicly available datasets
- A list of figures that have associated raw data
- A description of any restrictions on data availability

All data used for benchmarking and ENCODE analysis are available online at https://encodedcc.org/. Specific accessions and relevant metadata for each of the benchmarking experiments are available in tables S1 and S2. Accession IDs and metadata for all datasets from ENCODE analysis are available in table S3. Haplotype maps used for benchmarking and ENCODE analysis are available at https://github.com/naumanjaved/fingerprint_maps). RNA-seq data from CEPH/Utah Pedigree 1463 were downloaded from the Gene Expression Omnibus (https://www.ncbi.nlm.nih.gov/geo/) from series GSE56961, using accession IDs: SRR8505344, SRR8505340, SRR8505343, SRR1258219, SRR1258220, SRR1258218, and SRR8505347. 1000 Genome Phase 3 VCFs for hg19 and GRCh38 liftovers were obtained from ftp://ftp-trace.ncbi.nih.gov/1000genomes/ftp. Recombination maps for hg19 and GRCh38 liftovers were obtained from http://bochet.gcc.biostat.washington.edu/beagle/genetic_maps/.

Field-specific reporting

Please select the one below that is the best fit for your research. If you are not sure, read the appropriate sections before making your selection.

☒ Life sciences ☐ Behavioural & social sciences ☐ Ecological, evolutionary & environmental sciences

For a reference copy of the document with all sections, see nature.com/documents/nr-reporting-summary-flat.pdf

Life sciences study design

All studies must disclose on these points even when the disclosure is negative.

Sample size	No statistical tests were performed as part of this work and therefore no sample size calculation was necessary
Data exclusions	no data excluded
Replication	this work does not test any hypotheses so replication is irrelevant
Randomization	No statistical tests were performed as part of this work and therefore no randomization was necessary
Blinding	blinding is irrelevant for this work

Reporting for specific materials, systems and methods

We require information from authors about some types of materials, experimental systems and methods used in many studies. Here, indicate whether each material, system or method listed is relevant to your study. If you are not sure if a list item applies to your research, read the appropriate section before selecting a response.

Materials & experimental systems

n/a	Involved in the study	
☒	☐	Antibodies
☒	☐	Eukaryotic cell lines
☒	☐	Palaeontology
☒	☐	Animals and other organisms
☒	☐	Human research participants
☒	☐	Clinical data

Methods

n/a	Involved in the study	
☒	☐	ChIP-seq
☒	☐	Flow cytometry
☒	☐	MRI-based neuroimaging
